# The serine‐threonine kinase PIM3 is an aldosterone‐regulated protein in the distal nephron

**DOI:** 10.14814/phy2.14177

**Published:** 2019-08-08

**Authors:** Alessia Spirli, Lydie Cheval, Anne Debonneville, David Penton, Caroline Ronzaud, Marc Maillard, Alain Doucet, Johannes Loffing, Olivier Staub

**Affiliations:** ^1^ Department of Pharmacology & Toxicology University of Lausanne Lausanne Switzerland; ^2^ National Centre of Competence in Research “Kidney.ch” Lausanne Switzerland; ^3^ Centre de Recherche des Cordeliers INSERM, Sorbonne Universités, USPC, Université Paris Descartes, Université Paris Diderot, Physiologie Rénale et Tubulopathies Paris France; ^4^ Institute of Anatomy University of Zurich Zurich Switzerland; ^5^ Service of Nephrology Lausanne University Hospital (CHUV) Lausanne Switzerland

**Keywords:** Blood pressure, blood volume, kidney, steroid hormone

## Abstract

The mineralocorticoid hormone aldosterone plays a crucial role in the control of Na^+^ and K^+^ balance, blood volume, and arterial blood pressure, by acting in the aldosterone‐sensitive distal nephron (ASDN) and stimulating a complex transcriptional, translational, and cellular program. Because the complexity of the aldosterone response is still not fully appreciated, we aimed at identifying new elements in this pathway. Here, we demonstrate that the expression of the proto‐oncogene PIM3 (Proviral Integration Site of Moloney Murine Leukemia Virus 3), a serine/threonine kinase belonging to the calcium/calmodulin‐regulated group of kinases, is stimulated by aldosterone in vitro (mCCD_cl1_ cells), ex vivo (mouse kidney slices), and in vivo in mice. Characterizing a germline *Pim3* *^−^* *^/^* *^−^* mouse model, we found that these mice have an upregulated Renin‐Angiotensin‐Aldosterone System (RAAS), with high circulating aldosterone and plasma renin activity levels on both standard or Na^+^‐deficient diet. Surprisingly, we did not observe any obvious salt‐losing phenotype in *Pim3* KO mice as shown by normal blood pressure, plasma and urinary electrolytes, as well as unchanged expression levels of the major Na^+^ transport proteins. These observations suggest that the potential effects of the loss of the *Pim3* gene are physiologically compensated. Indeed, the 2 other family members of the PIM kinase family, PIM1 and PIM2 are upregulated in the kidney of *Pim3* *^−^* *^/^* *^−^* mice, and may therefore be involved in such compensation. In conclusion, our data demonstrate that the PIM3 kinase is a novel aldosterone‐induced protein, but its precise role in aldosterone‐dependent renal homeostasis remains to be determined.

## Introduction

The renin‐angiotensin‐aldosterone system (RAAS) plays an important role in the regulation of blood pressure, as evidenced by many genetic forms of human hypertension that map to the RAAS as well by the fact that blockers of this system are frequently used in the treatment of hypertension (Lifton et al. 2001a; Te Riet et al. 2015). The RAAS is activated under hypovolemic conditions and enhances the production of the mineralocorticoid hormone aldosterone in the *Zona glomerulosa* of the adrenal gland. In the kidney, aldosterone acts in the aldosterone‐sensitive distal nephron (ASDN, composed of the late part of the distal convoluted tubule (DCT2), the connecting tubule (CNT) and the collecting duct (CD)) (Loffing et al. 2001aa), where it stimulates Na^+^ reabsorption to restore Na^+^ balance, blood volume, and pressure (Yagi et al. 2013). At the level of an ASDN cell, aldosterone binds to the mineralocorticoid receptor (MR), and promotes its translocation into the nucleus. An intricate transcriptional and cellular program that involves the induction of genes containing MR response elements (MREs), such as the genes encoding *α*ENaC or Na^+^/K^+^‐ATPase, leads to increased Na^+^ reabsorption and K^+^ secretion (Staub et al. 2015). However, there are numerous other early‐ or long‐term induced transcripts, encoding regulatory proteins such as SGK1, GILZ, 14‐3‐3β or KS‐WNK1 (Chen et al. 1999; Naray‐Fejes‐Toth et al. 1999; Robert‐Nicoud et al. 2001; Naray‐Fejes‐Toth et al. 2004; Liang et al. 2006; Elvira‐Matelot et al. 2010), although this list is not exhaustive (Rossier et al. 2015). SGK1 is one of the most firmly established aldosterone‐induced protein in the kidney, and it has been proposed to act via phosphorylation of the ubiquitin‐protein ligase NEDD4‐2, thereby interfering with NEDD4‐2‐dependent inhibition of ENaC (Debonneville et al. 2001; Snyder et al. 2002). In 2003, Gumz and collaborators published a list of early aldosterone‐responsive transcripts (Gumz et al. 2003), which identified among other genes, a transcript encoding the Ser/Thr kinase PIM‐3. This kinase is a member of the PIM (Proviral Insertion site of Moloney murine leukemia virus) kinase family, consisting of three members (PIM1 to 3), and belonging to the calcium/calmodulin‐regulated kinase group (Feldman et al. 1998; Nawijn et al. 2011). These proteins are defined as proto‐oncogenes and are highly evolutionarily conserved in multicellular organisms. The three kinases are short‐lived proteins mainly regulated at the transcriptional level. They have an ATP‐binding pocket, an active site, a kinase domain, but no regulatory domains, therefore rendering them constitutively active (Narlik‐Grassow et al. 2014).

PIM‐3 was originally identified as a depolarization‐ or forskolin‐induced gene in rat PC12 cell, a pheocromocytoma cell line, and named KID‐1 (Feldman et al. 1998). Subsequently, it was renamed PIM‐3 due to its homology with the other PIM family members (Deneen et al. 2003). *Pim3* mRNA in humans is detected in several normal tissues such as brain, lung, spleen, placenta, skeletal muscle, peripheral blood leukocytes and kidney, but not in colon, thymus, liver or small intestine tissues (Eichmann et al. 2000). In mouse embryos, it is expressed in liver, kidney, lungs thymus, CNS, pancreas, stomach and intestinal epithelium. In some cases, PIM‐3 is not found in normal tissues, but becomes expressed in the initial phases of carcinogenesis (Mukaida et al. 2011). Several studies have also shown that the *Pim‐3* gene, contrary to the two other family members, is expressed in the mouse kidney (Mikkers et al. 2004; Cheval et al. 2011), and in isolated mouse DCT/CNT and cortical collecting duct (CCD) tubules (Pradervand et al. 2010). It is upregulated by aldosterone in an Inner Medullary Collecting Duct (IMCD) cell line (Gumz et al. 2003), and in dissected Outer Medullary Collecting Duct (OMCD) cells after 3 days of low K^+^ diet (Cheval et al. 2004). Moreover, the *Pim3* gene has been shown to be regulated by the circadian rhythm in several organs including the kidney (Yan et al. 2008; Bhargava et al. 2015). Despite its renal expression, no information is available about its function in this organ. In this study, we show the specific PIM3 renal expression along the nephron and its aldosterone regulation both in vitro (mCCD_cl1_ *),* ex vivo (kidney slices), and in vivo (Aldosterone Synthase KO mice, C57BL6 mice) experiments. Moreover, we characterized a *Pim‐3* KO mouse model, which we found to have an activated RAAS system, but a fully compensated renal salt phenotype. This compensated phenotype is most likely explained by a compensatory increase of the two other family members, PIM2 and PIM3.

## Material and Methods

### Mouse models

WT C57BL/6 male mice (8‐ to 12‐week old) were purchased from Charles Rivers whereas *Pim3* KO mice, were previously published (Mikkers et al. 2004) and kindly provided by Prof. A. Berns (Netherlands Cancer Institute, Amsterdam). *Pim3* heterozygous animals were in‐house crossed and WT and KO littermates obtained with the expected Mendelian ratio. All animals were maintained in a temperature‐controlled room, with free access to both food and drinking water, and adapted before each experiment to 12‐h light/dark cycle (normal or inverted) and experimental chow. Frozen kidneys from Aldosterone Synthase KO mice were provided by JL (Lee et al. 2005; Todkar et al. 2015). Experimental protocols were designed with respect to the Swiss Animal Welfare Act and approved by the veterinary administration of the Canton of Vaud, Switzerland.

### Genotyping

Genomic DNA extraction was performed from ear biopsies incubated at 56°C overnight in 0.2 mL of NID buffer (50 mmol/L KCl, 10 mmol/L Tris‐HCl, pH 8.3, 2 mmol/L MgCl_2_, 0.1 mg/mL Gelatin, 0.45% NP40, 0.45% Tween20) with 2 *μ*L of proteinase K (10 mg/mL). After heat inactivation of the proteinase K for 10 min at 96°C, 2 *μ*L of the DNA preparation were used for genotyping PCR using primers synthetized by Microsynth. For PIM3 genotyping four primers were used: PIM‐3 WT (F: 5′ CTG GAC CAA ATT GCT GCC CAC 3′), PIM‐3 WT (R: 5′ GGA TCT CTG GTT CAA GTA TCC 3′); PIM‐3‐LACZ (F: 5′ CGT CAC ACT ACG TCT GAA CG 3′), PIM‐3‐LACZ (R: 5′ CGA CCA GAT GAT CAC ACT CG 3′). The reaction yielded 400 bp and/or 550 bp DNA fragments corresponding to WT and KO alleles respectively, which are separated on 1.5% agarose gel for analysis.

### Experimental diets

Mice were challenged with experimental diets in powder or pellet produced by Ssniff Spezialdiäten GmbH, Soest, Germany, which differed for the content of Na^+^: normal or standard sodium diet (NSD, 0.2% Na^+^) and low sodium diet (LSD, 0.01% Na^+^), as described in (Faresse et al. 2012).

### Metabolic cages, plasma, and urinary electrolytes measurement

*Pim3* WT and KO male mice (8–12 week old), were placed individually in metabolic cages (Indulab, Cat. #3600M021) and fed on standard or low Na^+^ diet for different periods. After 3 days of adaptation, body weight, food and water intake were measured and urines collected every 24 h, for 4 days, at Zeitgeber time ZT 12 (beginning of the night period). At the end of the experiment (ZT 12) mice were irreversibly anesthetized with 0.8 mg Xylazine and 1 mg Ketamine in 0.9% NaCl per kg of body weight injected intraperitoneally. Blood was collected by retroorbital punction in Li^+^‐Heparine tubes (Sarstedt). The first 11 drops were used for plasma aldosterone measurements, while the remaining blood was used for plasma electrolytes analysis. The animals were then sacrificed by cervical dislocation and tissues were harvested, washed in ice‐cold PBS, quickly snap frozen in liquid nitrogen and kept at −80°C before analysis. Plasma and urinary Na^+^ and K^+^ were measured using a flame photometer (Cole‐Palmer Instruments, Vernon Hills, IL), whereas, urinary Ca^2+^ and creatinine analyses were performed by the Laboratory of Clinical Chemistry at the Lausanne Hospital (CHUV) using a Modular Analytics 118 System (Roche Diagnostics). Urinary osmolality was measured with an Advanced 2020 osmometer (advanced Instruments).

### Plasma aldosterone and plasma renin activity measurement

Plasma aldosterone levels were measured by RIA (Coat‐a‐Count; Diagnostic Products). To determine the plasma renin activity (PRA), mice kept on normal sodium diet were barely anesthetized with 50 mg/kg pentobarbital and blood collected in sodium/EDTA‐coated tubes (Sarstedt) after decapitation. PRA was determined according to a conventional RIA procedure. Both measures were performed in the laboratory of Marc Maillard (Service of Nephrology, CHUV).

### Telemetry

*Pim3* WT and KO mice, 8‐week old, were operated as described previously (Ronzaud et al. 2013). Mice were fed on standard or low‐sodium diet and after a 2‐week recovery period, signals were recorded for 9 sec every minute for 7 days. The mean arterial pressure (MAP) was calculated for day 7 as mean of 10‐h average (ZT1‐ZT11 for day period and ZT13‐ZT23 for night period) for each mouse.

### Microdissection of mouse renal tubules

Kidneys from WT C57BL/6 mice kept on standard or low‐sodium diet for 3 days were isolated and microdissected as previously described (Cheval et al. 2011). For mRNA analysis, RNA was extracted from 5 to 6 animals independently. For protein analysis, kidneys were perfused and microdissected as described previously (Christensen et al. 2011). For each tubular segment (PT, TAL, DCT/CNT, or CCD), tubules microdissected from 4 mice were pooled together (approximately 12 cm of tubule length per segment) and analyzed by immunoblotting).

### Kidney slices preparation

Kidney slices were prepared as previously described (Penton et al. 2016). Divided in groups, they were incubated with vehicle (ethanol) or 10 nmol/L aldosterone for 30 or 60 min. At the end of the treatment, the slides were homogenized in a buffer containing 200 mmol/L mannitol, 80 mmol/L Hepes and 41 mmol/L KOH and protein quantified for subsequent western blot analysis.

### Cell culture

Mouse cortical collecting duct clone 1 (mCCD_cl1_) cells (Gaeggeler et al. 2005) were used between passage 28 and 40 and cultured as previously described in plastic flasks for maintenance or in collagen‐coated filters for experimental procedures. After an overnight starvation in minimal medium, the cells on filters were stimulated for different time points with 50 nmol/L aldosterone, or for 90 min with aldosterone in combination with 10 *µ*mol/L eplerenone or 10 *µ*mol/L RU‐486; all chemicals were purchased from Sigma Aldrich. Transepithelial potential difference (*V*_te_: mV) and electrical resistance (*R*_te_: Ohm/cm^2^) were recorded under sterile conditions using EVOM^2^ Epithelial Volt/Ohm Meter (World Precision Instruments) and short circuit currents (Isc: *µ*A/cm^2^) were calculated as *V*_te_/*R*_te_.

### Quantitative real‐time PCR

Total RNA was extracted from cells, microdissected tubules or from ½ kidney homogenates using TRIzol (Invitrogen) and RNeasy MiniKit (QIAGEN). Reverse transcription was performed with Superscript II Reverse Transcriptase (Invitrogen) and cDNA was used for Real‐Time PCR performed with 7500 Fast Real‐Time PCR System (Applied Biosystems) and Taqman Gene Expression Assays (Applied Biosystems) for Pim3 (Mm 00446876_m1), Gapdh (Mm 99999915_g1), Rplp1 (Mm 02601846_g1), *α*ENaC (Mm00803386_m1), NCC (Mm00490213_m1), Pim1 (Mm00435712_m1), Pim2 (Mm004454579_m1), ROMK (Mm01173990_m1), Sgk1 (Mm00441380_m1). For microdissections, RNAs were extracted as previously described (Cheval et al. 2011) and, after first strand synthesis (transcriptor first strand cDNA synthesis kit) Real‐time PCR performed on a LightCycler (Roche) using LightCycler 480 SYBR Green I Master kit (Roche) according to the manufacturer’s protocols except for the reaction volume (10 *µ*L instead of 20 *µ*L) and the following primers: RPL26 F: 5′ GCT AAT GGC ACA ACC GTC 3′, RPL26 R: 5′ TCT CGA TCG TTT CTT CCT TGT AT 3′, PIM3 F: 5′ AAC ACT TTC TGC CTG GGA TG 3′, PIM3 R: 5′ AGG CAC TCA AAG CAA AGG AA 3′.

### Immunoblot analysis

For PIM3 analysis on microdissected tubules, the material was collected and resuspended directly in Sample Buffer ready to be loaded. On the other hand, frozen tissues were homogenized with polytron in extraction buffer (50 mmol/L Tris‐HCl, 1 mmol/L EDTA, 1 mmol/L EGTA, 0.27 mol/L Sucrose, pH 7.5) whereas mCCD_cl1_ cell lysates were performed in buffer containing: 50 mmol/L HEPES, 150 mmol/L NaCl, 1 mmol/L EGTA, 10% glycerol, 1% triton X100. All buffers contained Complete^TM^ protease inhibitor cocktail and phosphatase inhibitor PhosSTOP (Roche). After sonication, extracts were centrifuged at 10,000*g* for 10 min (kidney homogenates) or 22,000*g* for 15 min (cell lysates), and protein concentration was assessed by Bradford quantification (Brunschwig). 20 *µ*g of protein were migrated in Mini‐PROTEAN^®^ TGX Stain‐Free^TM^ gels (BIO‐RAD) and transferred to nitrocellulose membranes. Western blottings were performed using the following antibodies: anti‐PIM3 (1:500, Cell Signaling), anti‐actin and ‐tubulin (1:1000, Sigma Aldrich), anti‐NHE3 (Merck; 1:10), anti‐pNKCC (Mutig et al. 2007) (1:1000, provided by Prof. S. Bachmann), anti‐NKCC2 (1:1000, (Wagner et al. 2008)), anti‐NCC (1:8000; Chemicon), anti‐pNCC (1:1000; (Ronzaud et al. 2015).

### Statistical analysis

All values are expressed as mean ± Standard Deviation (SD). Data were analyzed with GraphPad PRISM version 7.03 software for windows and Student’s *t* test was used when only two groups were considered. In the presence of three or more groups one‐ or two‐way ANOVA followed by post hoc Bonferroni’s test for multiple correction were applied. A *P* value of less than 0.05 was considered significant.

## Results

### PIM3 is ubiquitously expressed along the nephron

**Figure 1:**
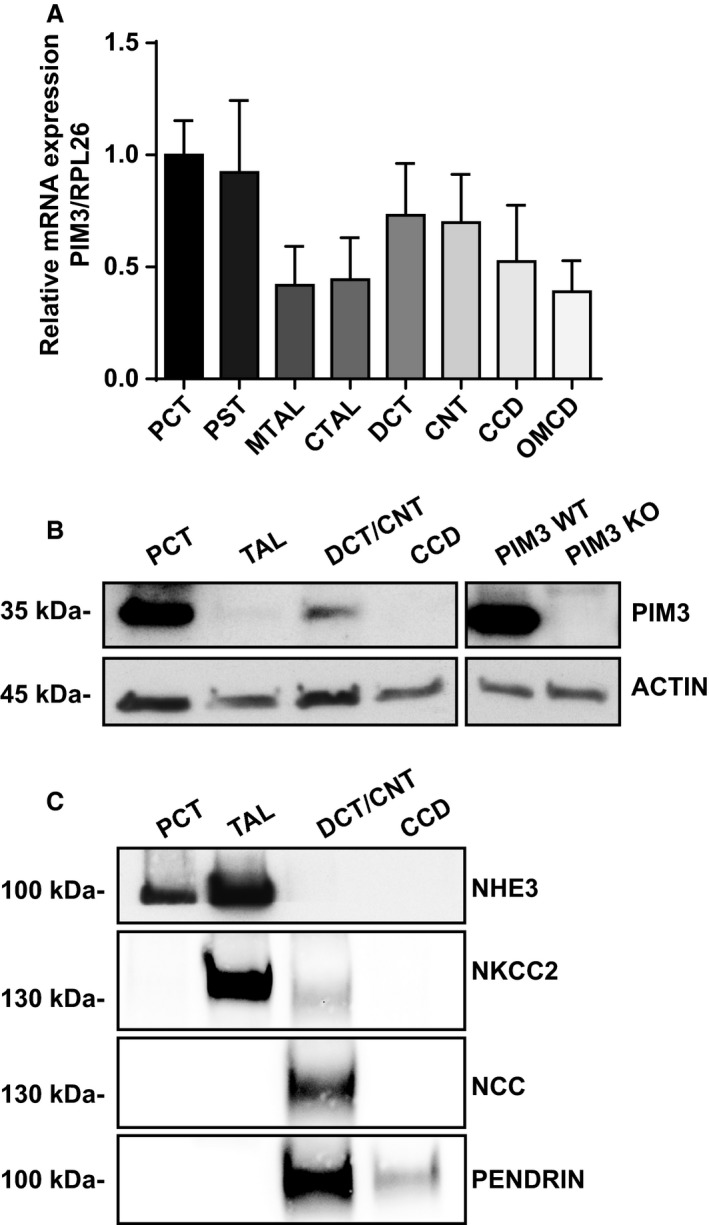
PIM3 renal expression. (A) Quantification by RT‐PCR of Pim3 mRNA in microdissected mouse renal tubules normalized to Rpl26 (*n* = 5, normal sodium diet). Values are expressed as mean ± SD. (B) Representative western blot of microdissected mouse renal tubules and total kidney lysates probed with anti PIM3 or ACTIN antibodies (pool of *n* = 3 for PCT; *n* = 5 for TAL, DCT/CNT, and CCD, normal sodium diet). (C) Representative Western blot analysis of microdissected mouse renal tubules probed with NHE3 as marker of PCT and TAL, NKCC2 for TAL, NCC for DCT/CNT and PENDRIN for the distal nephron.

It has been reported that the *Pim3* mRNA is detectable in mouse total kidney (Mikkers et al. 2004) and renal cell lines (Gumz et al. 2003; Pradervand et al. 2010). To characterize in detail PIM3 expression along the nephron, we performed quantitative RT‐PCR on microdissected segments of nephrons from adult mice kept on a standard diet as described previously (Cheval et al. 2011). We found *Pim3* mRNA ubiquitously expressed along the nephron, but more prominent in proximal (PCT: proximal convoluted tubule and PST: proximal straight tubule) as well as in distal (DCT, CNT, CCD) tubules (Fig. 1A). The same pattern was found at the protein level in western blot analysis of microdissected mouse renal tubules (Fig. 1B), where PIM3 is expressed as a 35 kDa protein, and completely lost in total kidney lysate of a germline *Pim3* KO mouse. Western blot of segment‐specific markers validated the quality of the microdissection (Fig. 1C). These data confirmed the renal expression of the Ser/Thr kinase PIM3 and its specific localization along the nephron. In the CCD, the PIM3 protein was not detectable, suggesting that in this segment the basal levels of the kinase might be very low.

### *PIM3* is regulated in vitro by aldosterone via the mineralocorticoid receptor

**Figure 2:**
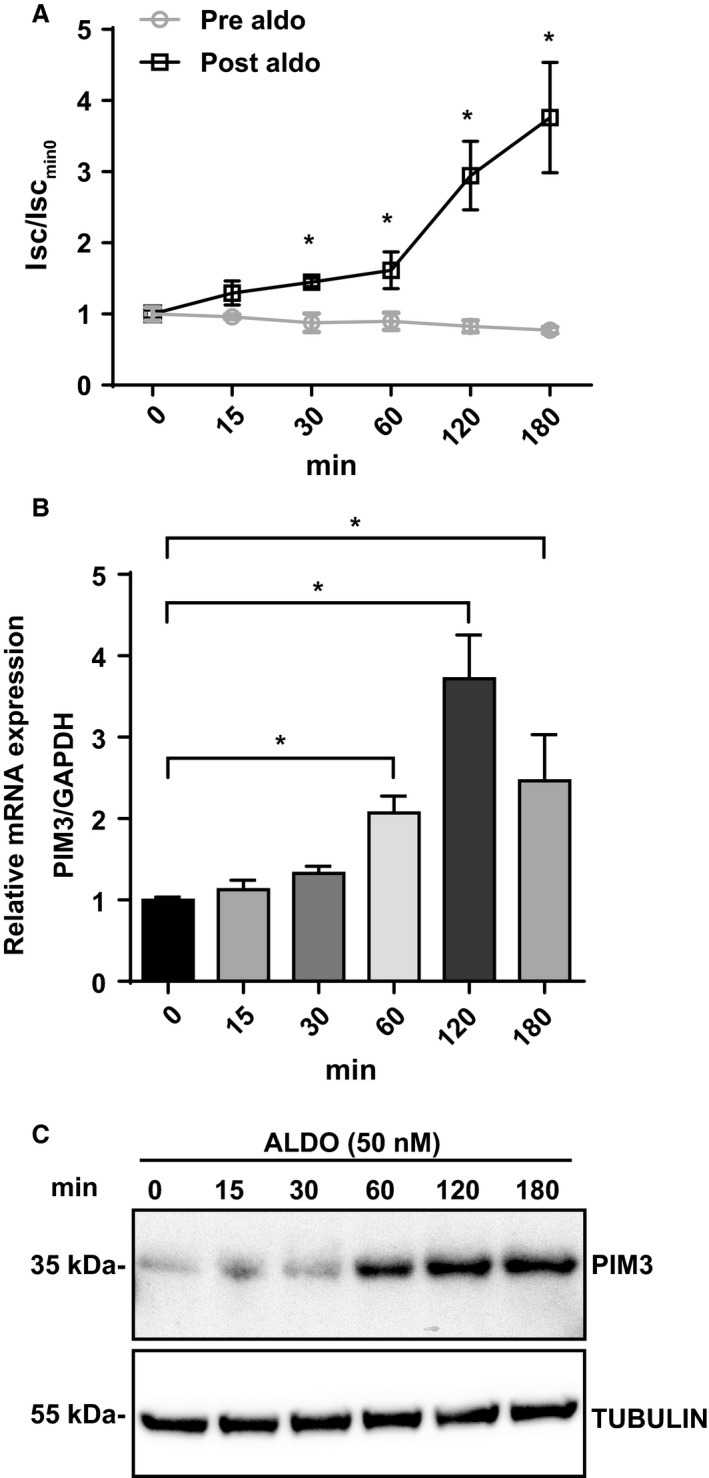
PIM3 is regulated in vitro by aldosterone. mCCDcl1 cells were treated with 50 nmol/L aldosterone for the indicated time points. (A) The Short Circuit Current (Isc) was calculated before and after aldosterone stimulation (*n* = 3), Isc of each time point was normalized to that of time zero. *P < 0.05, versus time zero, one‐way ANOVA (post hoc Bonferroni’s test) (B) TaqMan analysis of Pim3 mRNA normalized over Gapdh (*n* = 3). *P < 0.05, one‐way ANOVA (post hoc Bonferroni’s test). Values are expressed as mean ± SD. (C) Representative Western blot of cell lysates probed with anti PIM3 and TUBULIN antibodies.

**Figure 3:**
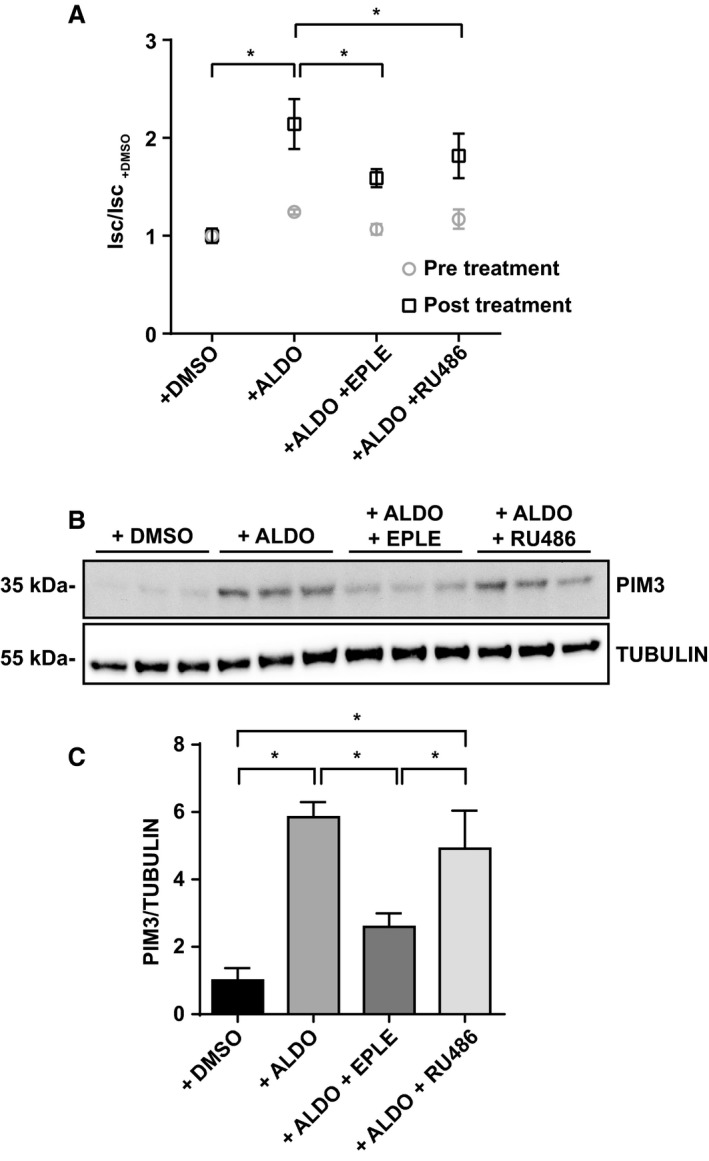
PIM3 is regulated in vitro by aldosterone through the mineralocorticoid receptor (MR). mCCDcl1 cells were treated with 50 nmol/L aldosterone alone or in combination with 10 *μ*mol/L eplerenone (MR inhibitor) or 10 *μ*mol/L RU‐486 (GR inhibitor) for 90 min. (A) Short circuit current was calculated before and after treatment (*n* = 3), Isc of each condition was normalized to that of +DMSO. *P < 0.05, one‐way ANOVA (post hoc Bonferroni’s test). (B) Western blot analysis of PIM3 and TUBULIN and relative densitometric analysis (C) (*n* = 3). *P < 0.05, one‐way ANOVA (post hoc Bonferroni’s test). Values are expressed as mean ± SD.

As it was shown previously that *Pim3* mRNA expression is stimulated by supraphysiological levels of aldosterone (1 *μ*mol/L for 1 h) in IMCD cells (Gumz et al. 2003), we wondered if PIM3 is regulated by aldosterone in the CCD. We took advantage of a well‐established mouse cortical collecting duct cell line that was previously shown to be regulated by physiological concentrations of aldosterone (mCCD_cl1_) (Gaeggeler et al. 2005). We stimulated mCCD_cl1_ cells with 50 nmol/L aldosterone for different lengths of time. *Pim3* mRNA and protein analysis was performed after having measured the short circuit current, as a control for stimulation of electrolytes transport (Fig. 2A). As compared to time zero, the *Pim3* mRNA expression was significantly upregulated starting at 1 h of aldosterone treatment (Fig. 2B); consistently, we observed an increase in PIM3 protein levels at the same time points (Fig. 2C). Because the effects of aldosterone in the kidney can be mediated via either the mineralocorticoid receptor (MR) or the glucocorticoid receptor (GR), hormonal stimulation was performed alone or in combination with two receptors antagonists: eplerenone (10* µ*mol/L) and RU‐486 (10* µ*mol/L), respectively. As shown in Figure 3A, the aldosterone‐induced transepithelial short circuit current (I_SC_) was reduced in the presence of either antagonist, demonstrating that aldosterone acts via both receptors under these conditions. However, only in the presence of eplerenone, and not RU‐486, aldosterone‐dependent induction of the PIM3 protein was significantly blunted (Fig. 3B and C), suggesting that in CCD cells, PIM3 expression is regulated by aldosterone and this effect occurs primarily via MR.

### Aldosterone regulates PIM3 in the kidney ex vivo and in vivo

**Figure 4:**
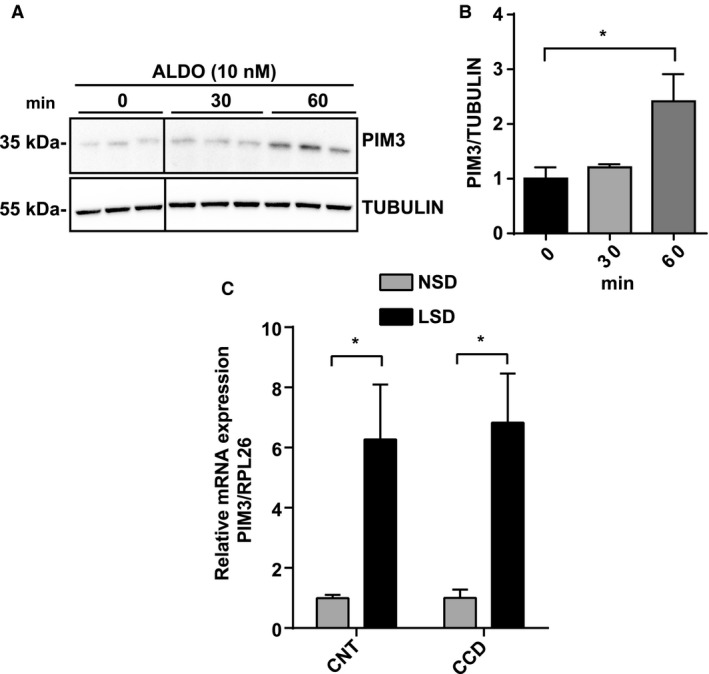
Aldosterone regulates PIM3 in the kidney ex vivo and in vivo. Kidney slices were incubated for the indicated time points with 10 nmol/L aldosterone and subjected to western blot analysis (A) for PIM3 and TUBULIN for normalization (*n* = 3). Signals were analyzed by densitometry (B), *P < 0.05, one‐way ANOVA (post hoc Bonferroni’s test). Values are expressed as mean ± SD. Lanes loaded in the same gel but not contiguous are indicated by black lines. (C) Mice were administered a normal (0.2% Na^+^) or a low sodium (0.01% Na^+^) diet for 3 days and microdissected CNT (*n* = 6) and CCD (*n* = 5) from both conditions were analyzed for Pim3 or Rpl26 by quantitative RT‐PCR, *P < 0.05, two‐way ANOVA (post hoc Bonferroni’s test).

**Figure 5:**
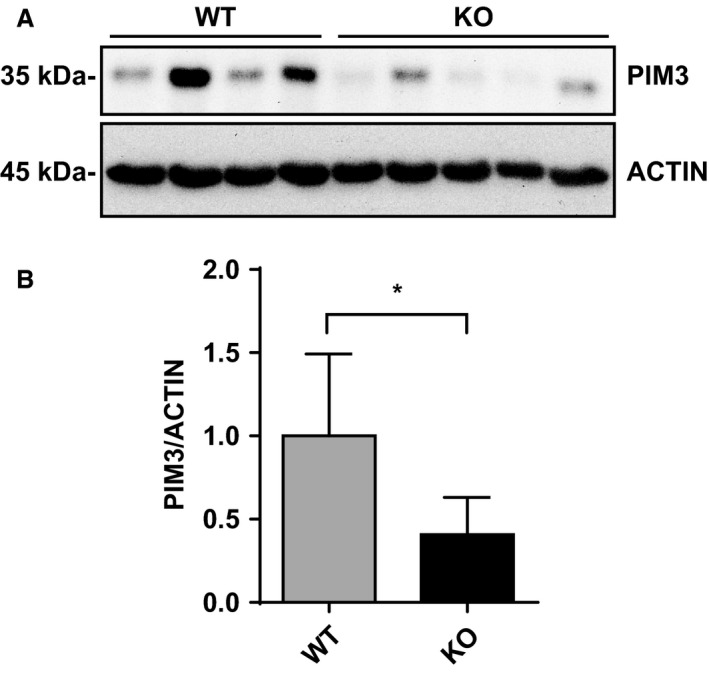
PIM3 is downregulated in aldosterone synthase KO mice. Total kidney lysates from Aldosterone Synthase WT (*n* = 4) and KO mice (*n* = 5) were subjected to Western blot (A) for PIM3 and ACTIN and signals were analyzed by densitometry (B), *P < 0.05, Student’s *t* test. Values are expressed as mean ± SD.

We further validated the aldosterone‐dependent PIM3 regulation under more physiological conditions. First, we performed an ex vivo time course experiment, similar to that done in mCCD_cl1_ cells using mouse kidney slices obtained as described previously (Penton et al. 2016). The preparations were incubated with 10 nmol/L aldosterone for different time points; immunoblot analysis revealed that the PIM3 protein level was significantly increased after 1 h of hormonal stimulation (Fig. 4A and B), similar to what we observed in the mCCD_cl1_ cells. To demonstrate the same regulation in vivo, C57BL/6 mice were fed with normal (NSD) or low sodium (LSD) diet for 3 days and microdissected CNT and CCD segments were analyzed by quantitative RT‐PCR. *Pim3* mRNA was significantly upregulated in the condition of LSD in both portions of the nephron (Fig. 4C). We then wondered if PIM3 was downregulated in a mouse model deficient for the aldosterone synthase (AS) enzyme described previously (Lee et al. 2005; Todkar et al. 2015). Immunoblotting of total kidney lysates of AS WT and KO mice showed that in the absence of aldosterone PIM3 is significantly reduced, suggesting an important role of this hormone in the control of PIM3 renal expression (Fig. 5A and B). In summary, these findings suggest that in the ASDN, the Ser/Thr kinase PIM3 is regulated by the mineralocorticoid hormone aldosterone.

### *Pim3* KO mice show increased plasma aldosterone and renin activity but normal blood pressure

**Figure 6:**
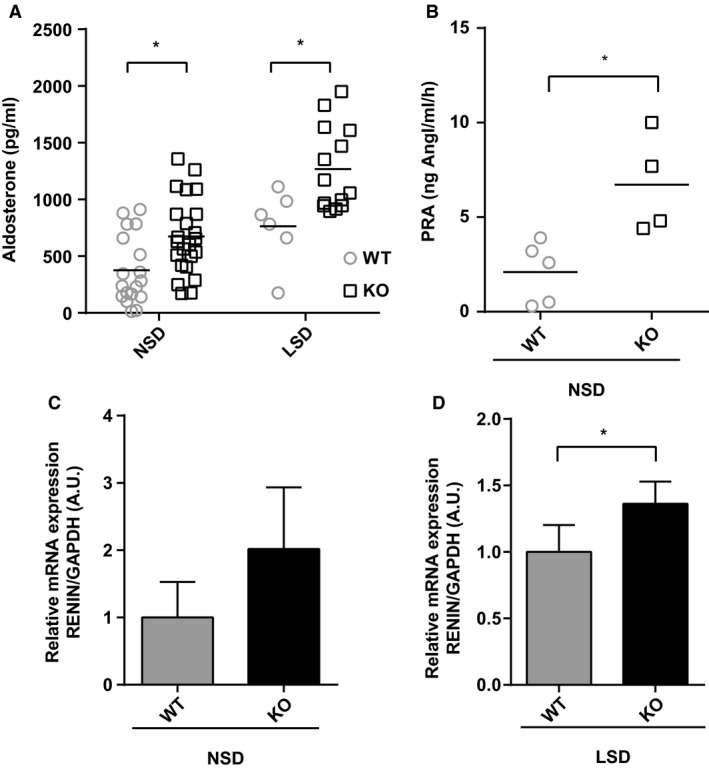
*Pim3* KO mice show increased plasma aldosterone and plasma renin activity associated with higher renin mRNA. (A) Plasma aldosterone levels in Pim3 WT (*n* = 18–6) and KO (*n* = 23–14) mice after 7 days of normal (NSD) or low (LSD) sodium diet. *P < 0.05, two‐way ANOVA (post hoc Bonferroni’s test). (B) Plasma renin activity (PRA) measured in Pim3 WT (*n* = 5) and KO (*n* = 4) mice on normal sodium diet. *P < 0.05, Student’s *t* test. (C and D) TaqMan analysis of renin mRNA in total kidney RNA extraction from mice kept on normal or low sodium diet and normalized to GAPDH, *n* = 4–5, *P < 0.05, Student’s *t* test. Values are expressed as mean ± SD.

**Figure 7:**
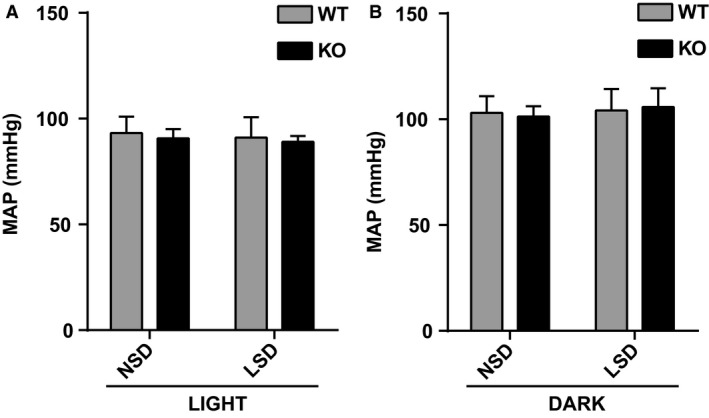
Pim3 WT and KO mice show similar blood pressure. (A and B) Mean arterial pressure (MAP), 10‐h average, of *Pim3* WT (*n* = 5) and KO (*n* = 5–9) mice after 7 days of NSD or LSD, during inactive (LIGHT) and active (DARK) phase. Data were analyzed with two‐way ANOVA (post hoc Bonferroni’s test), no significant changes were found between genotypes. Values are expressed as mean ± SD.

We next explored the physiological consequences of PIM3 deletion in mice using a germline *Pim3* KO model previously created for the generation of a triple KO of all the PIM kinases (Mikkers et al. 2004). WT and KO animals were fed with standard or low Na^+^ diet (LSD) for 7 days and placed in metabolic cages for urinary and plasma parameter analysis. As can be seen in Table I, there were no differences in food and water intake and urinary volume production between genotypes. While there were no differences in plasma or urinary Na^+^ and K^+^ under both diets, we observed some small changes in the hematocrit, osmolality and Ca^2+^ excretion (Table 1). Interestingly, *Pim3* *^−^* *^/^* *^−^*‐deficient mice displayed elevated plasma renin activity (PRA) (Fig. 6B), elevated *renin* mRNA levels in the total kidney (Fig. 6C) and higher plasma aldosterone levels when compared to controls (Fig. 6A). These observations suggest an over‐activation of the Renin‐Angiotensin‐Aldosterone System (RAAS) in mice lacking the PIM3 kinase, which likely is a compensatory mechanism to counteract hypovolemia. However, radiotelemetric analysis of the blood pressure did not show any difference between the genotypes (Fig. 7).

**Table 1:** Metabolic parameters in Pim3 WT and KO mice on standard (0.2%Na) and low (0.01% Na) sodium diet (7 days, 24‐hour urinary collection)

	Standard diet (Day 7)		Low sodium diet (Day 7)	
	PIM3 WT	PIM3 KO	PIM3 WT	PIM3 KO
Body weight (g)	26.64 (1.90)	25.08* (1.24)	26.68 (2.03)	24.64* (1.80)
Food intake (g/g BW)	0.16 (0.02)	0.16 (0.05)	0.15 (0.02)	0.15 (0.01)
Water intake (ml/g BW)	0.11 (0.01)	0.12 (0.01)	0.13 (0.02)	0.13 (0.03)
Urine				
Urine volume (ml/g BW)	0.03 (0.01)	0.04 (0.01)	0.02 (0.01)	0.03 (0.01)
Osmolality (mmol/Kg H_2_0)	5321.50 (856.85)	4436.36* (581.91)	5194 (1532.52)	4170 (552.43)
Na^+^ excretion (μmol/g BW)	5.34 (1.61)	5.77 (1.89)	0.08 (0.04)	0.09 (0.04)
Na7Cr (mmol/mmol)	56.81 (11.42)	59.75 (20.52)	0.93 (0.67)	0.71 (0.38)
K^+^ excretion (μmol/g BW)	19.29 (4.23)	19.18 (5.96)	14.25 (8.10)	17.41 (5.54)
K^+^/Cr (mmol/mmol)	197.16 (42.12)	197.79 (59.22)	119.09 (36.70)	138.67 (39.48)
Ca^2+^ excretion (μmol/g BW)	0.08 (0.04)	0.13* (0.06)	0.09 (0.05)	0.14 (0.07)
Ca^2+^/Cr (mmol/mmol)	0.84 (0.22)	1.27* (0.44)	0.87 (0.35)	1.19 (0.42)
Plasma				
Hematocrit (%)	41.55 (1.75)	44 .00* (1.86)	43.13 (2.17)	45.00 (2.68)
Na^+^ (mM)	138.8 (14.89)	140.2 (13.73)	146.06 (2.31)	148.02 (2.86)
K^+^ (mM)	4.73 (0.45)	4.77 (0.46)	6.54 (0.68)	6.02 (0.63)
Ca^2+^ (mM)	ND	ND	ND	ND

WT mice: *n* = 10–19 (NSD) and *n* = 5–8 (LSD). KO mice: *n* = ll–21 (NSD) and *n* = 10–12 (LSD). *P < 0.05, KO versus WT, Student's *t* test within each diet. Values are expressed as mean ± SD. Standard deviation indicated in parentheses.

### Expression and function of *α*ENaC, NCC, and NKCC2 in *Pim3* WT and KO mice after 7 days of normal or low sodium diet

**Figure 8:**
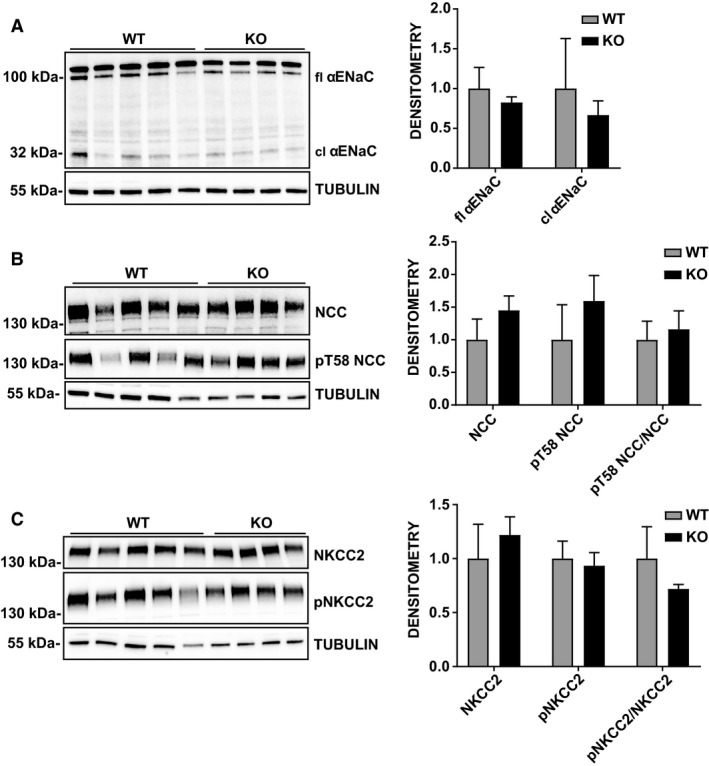
Expression of *α*ENaC, NCC and NKCC2 in Pim3 WT and KO mice kept under normal sodium diet for 7 days. Kidneys were harvested after metabolic cages experiments and protein extractions were blotted for the principal sodium channels or transporters. Representative Western blots and densitometric analyses of *α*ENaC (A), NCC and pT58NCC (B), NKCC2 and pNKCC2 (C) normalized to TUBULIN. *n* = 5 WT, *n* = 4 KO. Two‐way ANOVA (post hoc Bonferroni’s test) KO versus WT, no significant changes were found between genotypes. Values are expressed as mean ± SD.

**Figure 9:**
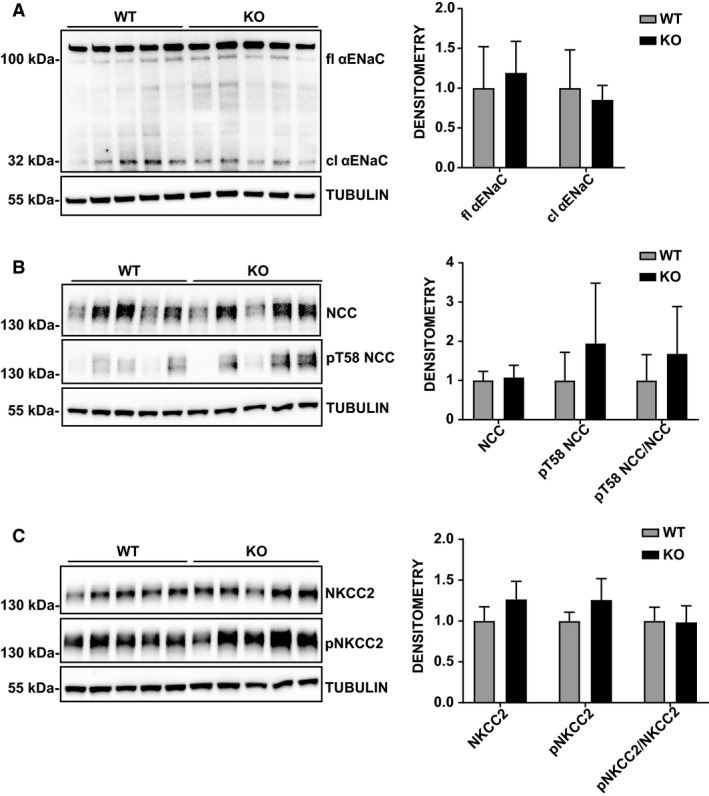
Expression of *α*ENaC, NCC and NKCC2 in Pim3 WT and KO mice kept under low sodium diet for 7 days. Kidneys were harvested after metabolic cages experiments and protein extractions were blotted for the principal sodium channels or transporters. Representative Western blots and densitometric analyses of *α*ENaC (A), NCC and pT58NCC (B), NKCC2 and pNKCC2 (C) normalized to TUBULIN. *n* = 5 WT, *n* = 5 KO. Two‐way ANOVA (post hoc Bonferroni’s test) KO versus WT, no significant changes were found between genotypes Values are expressed as mean ± SD.

In a compensated system, a potential defect, for example in a specific segment of the nephron, may be counterbalanced by another activity in another part of the nephron. Therefore, we investigated whether PIM3 deletion does effect the expression and function of the principal Na^+^ channels and transporters. First, we evaluated the expression of *α*ENaC, NCC, and NKCC2 in total kidney lysates from Pim3 WT and KO mice on normal or low‐Na^+^ diet. As shown in Figures 8 and 9, no changes in the expression and phosphorylation of these transporters were found between WT and KO mice, suggesting that the suppression of PIM3 does not affect these major sodium transporters in the kidney.

### *Pim1, Pim2,* and *Sgk1* mRNA are upregulated in *Pim3* KO mice

**Figure 10:**
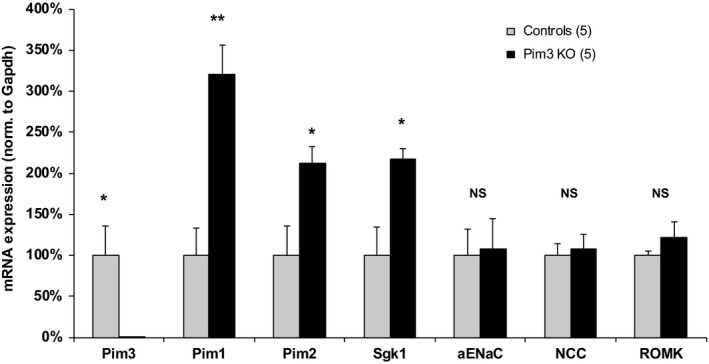
*Pim1, Pim2,* and *Sgk1* mRNA are upregulated in *Pim3* KO mice. Wild‐type and *Pim3* KO mice were kept for 7 days under a low sodium diet. Kidneys were harvested and *Pim3, Pim1, Pim2, Sgk1, αENaC, NCC,* and *ROMK* mRNA was quantified by quantitative real‐time PCR and normalized to *Gapdh*. Unpaired *t* test: **P < 0.01, *P < 0.05, NS: not significant.

Because the *Pim3* KO mice appear to be fully compensated, we wondered if PIM1 and/or PIM2 are upregulated in these mice. Moreover, the elevated plasma aldosterone found in *Pim3* KO mice, motivated us to investigate also the expression of *Sgk1, αENaC, NCC,* and *ROMK* mRNA. Mice were kept under a low Na^+^ diet for 7 days, to stimulate circulating aldosterone levels. As can be seen in Figure 10, *Pim1, Pim2*, as well as *Sgk1* mRNA were strongly upregulated in these mice, whereas *αENaC*, *NCC,* and *ROMK* remained at the same level as in wild‐type mice. We conclude that PIM1 and PIM2, likely in concert with SGK1, are able to counterweigh the deletion of PIM3.

## Discussion

To date, the primary role of Ser/Thr PIM kinases has been shown to be related to tumorigenesis and they are indeed considered as proto‐oncogenes (Nawijn et al. 2011; Narlik‐Grassow et al. 2014). Physiologically, these stress‐response kinases are broadly expressed in several organs and induced by cytokines, growth factors, hormones, and mitogenic stimuli (Xu et al. 2014). PIM3 is strongly expressed in human and mouse kidney, but its role and regulation within this organ has not been investigated. Our study was motivated by a study of Gumz et al. who showed that *Pim3* mRNA is upregulated by aldosterone (1* μmol*/L) in IMCD cells (Gumz et al. 2003).

Our analysis on the distribution along nephron shows that PIM3 is expressed both at the mRNA and protein level in all the segments, with higher expression both in the proximal tubule (PCT‐PST), as well as in the distal nephron (Fig. 1). These findings are consistent with previous large scale studies (Cheval et al. 2004; Pradervand et al. 2010; Cheval et al. 2011; Lee et al. 2015; Chen et al. 2017); interestingly *Pim3* mRNA levels are elevated in intercalated cells, as evidenced by single‐cell analysis by Chen *et al.* whereas relatively little is detected in principal cells (Chen et al. 2017). This may be explained by the requirement of aldosterone dependent stimulation of PIM3 expression in the principal cells. We cannot exclude that PIM3 is also expressed in other regions of the kidney (such as connective tissue or blood vessels), but due to the unavailability of specific PIM3 antibodies working for immunofluorescence analysis we could not address such questions.

Our findings, both in vitro and in vivo, clearly demonstrate that PIM3 is a physiological aldosterone‐induced transcript/protein. This is supported by experiments done in mCCD_cl1_ cells, showing that 50 nmol/L aldosterone stimulates PIM3 expression at the mRNA/protein level, that this stimulation can be inhibited with MR, but not GR antagonists, that aldosterone can stimulate PIM3 expression in ex vivo treated kidney slices, and that *Pim3* mRNA is increased in animals that were kept under low sodium diet, or stimulated with aldosterone, either in CNT or CCD segments, or in the total ASDN. Moreover, animals devoid of aldosterone‐synthase show a lower expression of PIM3. It comes as a surprise that changes in PIM3 expression can be observed in extracts of kidney slice preparations, or of total kidneys (Figs. 4 and 5), in view of the strong expression of PIM3 in the proximal tubule, where we do not expect an aldosterone response. However, changes in *Pim3* mRNA levels in microdissected tubules (CNT or CCD) upon a low Na^+^ diet are quite strong (up to 7fold) and may therefore be observed in total kidneys extracts. We cannot exclude that PIM3 is also regulated in the proximal tubule, for example by aldosterone‐related effects, as reported previously (Todd‐Turla et al. 1993; Leite‐Dellova et al. 2018). There are numerous proteins/transcripts that have been described to be regulated by aldosterone, including well characterized ones such as SGK1, *α*ENaC, or GILZ (for a review see (Rossier et al. 2015)). Due to its functional nature (kinase), and the early aldosterone induction and short half‐life, PIM3 may be functionally more similar to SGK1 (Loffing et al. 2006) than to ENaC (Mick et al. 2001; Loffing et al. 2001ab) or GILZ (Robert‐Nicoud et al. 2001). Both PIM3 and SGK1 are Ser/Thr kinases, and are induced in cortical collecting duct cells within 1 h of aldosterone stimulation, suggesting a potential‐related function in the regulation of Na^+^ and K^+^ homeostasis in the distal nephron. Nevertheless, the strength of SGK1 induction in the CCD, both in vitro and in vivo, appears to be much higher than that of PIM3, suggesting that if the two kinases are regulating related aldosterone‐mediated signaling pathways, PIM3 may have a less pronounced role as does SGK1. In order to gain a primary hint that PIM3 may be involved in aldosterone‐dependent regulation of electrolytes, blood volume, or blood pressure, we studied in wild‐type and *Pim3* KO mice aldosterone, plasma renin activity and mRNA expression in the kidney. Indeed, similarly to *Sgk1* KO (Wulff et al. 2002; Fejes‐Toth et al. 2008; Faresse et al. 2012), *Scnn1a,b,c* KO (Perrier et al. 2016; Boscardin et al. 2017, 2018) and *Nr3c2* KO (Canonica et al. 2016; Terker et al. 2016), but not *Tsc22d3 (GILZ)* KO mice (Rashmi et al. 2017), these animals displayed higher aldosterone levels, accompanied by elevated PRA and renin mRNA expression in the kidney.

As outlined in the introduction, aldosterone is either increased when kalemia is elevated, or if there is hypovolemia, during which aldosterone secretion would be stimulated via the RAAS. Our findings that both renin and aldosterone are increased in the *Pim3* KO clearly indicate that salt‐losing and hypovolemia must be at the origin of the misregulation of renin and aldosterone. However, the *Pim3* WT and KO mice that were challenged with standard and low Na^+^ diet did not reveal significant differences. The urinary electrolytes excretion was similar between genotypes except for a small difference in Ca^2+^, which was higher in KO mice on standard diet. Due to a recognized link between calcium and sodium reabsorption in the kidney (Moor and Bonny, 2016), a higher calcium secretion could be a secondary effect of a downregulation of proteins like NKCC2, or an upregulation of NCC (Ronzaud et al. 2013); however, the expression of these 2 transporters was not different between genotypes. We cannot exclude extra renal effects, such as elevated calcium reabsorption in the intestine, or a defect at the bone levels, which may cause indirectly the elevated calcium excretion, as observed in *Usp2* KO mice (Pouly et al. 2016). Moreover, urinary osmolality was lower in *Pim3* KO mice despite no changes in urinary volume, electrolytes content and AQP2 expression (not shown). At the plasma level, the higher hematocrit and higher plasma aldosterone and PRA levels led us to presume that *Pim3* KO mice have a problem of volume regulation. However, blood pressure measurements revealed no differences between WT and KO mice under both normal and low Na^+^ diets, and we detected no differences in the expression of various Na^+^ transporters or channels along the nephron. These findings suggest that these mice can compensate for the loss of PIM3. Indeed, when analyzing the expression levels of the 2 other PIM kinase members, PIM1 and PIM2, we found that the corresponding mRNAs in the kidneys of *Pim3* KO mice were strongly upregulated. Together with the expected elevated SGK1 expression (due to increased aldosterone), we consider it as reasonable that PIM1 and PIM2 elevated expression are at the basis of the compensation of the phenotype. We are currently investigating the signaling pathways that may be affected by aldosterone and PIM3 in the kidney.

## Conflict of Interest

None declared.
